# The complete mitochondrial genome of *Parapenaeopsis hungerfordi* (Decapoda: Penaeidae)

**DOI:** 10.1080/23802359.2018.1527194

**Published:** 2018-10-30

**Authors:** Shengping Zhong, Yanfei Zhao, Qin Zhang

**Affiliations:** Key Laboratory of Marine Biotechnology, Guangxi Institute of Oceanology, Beihai, China

**Keywords:** Mitochondrial genome, *Parapenaeopsis hungerfordi*, Decapoda

## Abstract

The dog shrimp, *Parapenaeopsis hungerfordi*, is an important commercial fishery species throughout the Indo-west Pacific. However, the resource of Penaeid shrimps, including *P. hungerfordi* decreased steadily in the recent years. Moreover, adequate mitogenome information about *P. hungerfordi* is still missing. In this study, we report the complete mitochondrial genome sequence of *P. hungerfordi*. The mitogenome has 15,952 base pairs (66.7% A + T content) and is made up of a total of 37 genes (13 protein-coding, 22 transfer RNAs, and 2 ribosomal RNAs), and a control region. The complete mitogenomes of *P. hungerfordi* will provide useful genetic information for future phylogenetic and taxonomic classification of Penaeidae.

Penaeid shrimps (Decapoda: Penaeidae) are an important resource in crustacean fisheries, which are also generally considered to be a primitive group of decapod crustaceans (Lavery et al. 2004). The dog shrimp, *P. hungerfordi* is an important commercial fishery species throughout the Indo-west Pacific, which inhabits open sea areas with sandy or muddy bottom at depths of 5–13 m. However, due to overexploitation and the breed descending in past decades, the resource of Penaeid shrimps, including *P. hungerfordi* decreased steadily in recent years (Samphan et al. 2015). The stock enhancement programs needs to be conducted for recovery wild populations of *P. hungerfordi*. The complete mitochondrial genome is a useful molecular technique for genetic structure assessment, however, in spite of its economic and ecological importance, adequate Mitogenome information about *P. hungerfordi* is still missing (Mao et al. 2016). Here, we report the complete mitochondrial genome sequence of *P. hungerfordi*, which will be an important genetic resource to assist in population genetics and resource management of *P. hungerfordi*.

A tissue samples of *P. hungerfordi* from five individuals were collected from GuangXi province, China (Beihai, 21.453738 N, 109.324589 E), and the whole body specimens (#GQ0321) were deposited at Marine biological Herbarium, Guangxi Institute of Oceanology, Beihai, China. The total genomic DNA was extracted from the muscle of the specimens using an SQ Tissue DNA Kit (OMEGA, Guangzhou, China) following the manufacturer’s protocol. DNA libraries (350 bp insert) were constructed with the TruSeq NanoTM kit (Illumina, San Diego, CA) and were sequenced (2 × 150 bp paired-end) using HiSeq platform at Novogene Company, China. Mitogenome assembly was performed by MITObim (Hahn et al. 2013). Complete mitogenome of *P*. *hardwickii* (GenBank accession number: NC_030277) was chosen as the initial reference sequence for MITObim assembly. Gene annotation was performed by MITOS (Bernt et al. 2013).

The complete mitogenome of *P. hungerfordi* was 15,952 bp in length (GenBank accession number: MG873460), and contains the typical set of 13 protein-coding, 22 tRNA and 2 rRNA genes, and a putative control region. The overall base composition of the mitogenome was estimated to be A: 34.4%, T: 30.1%, C: 23.4%, and G: 12.0%, with a high A + T content of 64.5%, which is similar, but slightly lower than *Melicertus latisulcatus* (66.7%) (Zhong et al. 2018). *Parapenaeopsis* and *Metapenaeus* shared very similar morphological characters, which have symmetric thelycum but distinct longitudinal groove along the dorsal carapace. The result of phylogenetic tree of 14 species (including other 13 species from Penaeidae in NCBI) also indicated the close relationship between *Parapenaeopsis* and *Metapenaeus* (Figure 1), which is consistent with the phylogenetic analyses of Penaeidae based on mitochondrial 16S rRNA gene (Chan et al. 2008). Our mitogenome data supported the sister relationship of *Parapenaeopsis* and *Metapenaeus.* The complete mitochondrial genome sequence of *P. hungerfordi* will be useful genetic information for further phylogenetic and comparative mitogenome studies of the family Penaeidae and related families.

**Figure 1.**
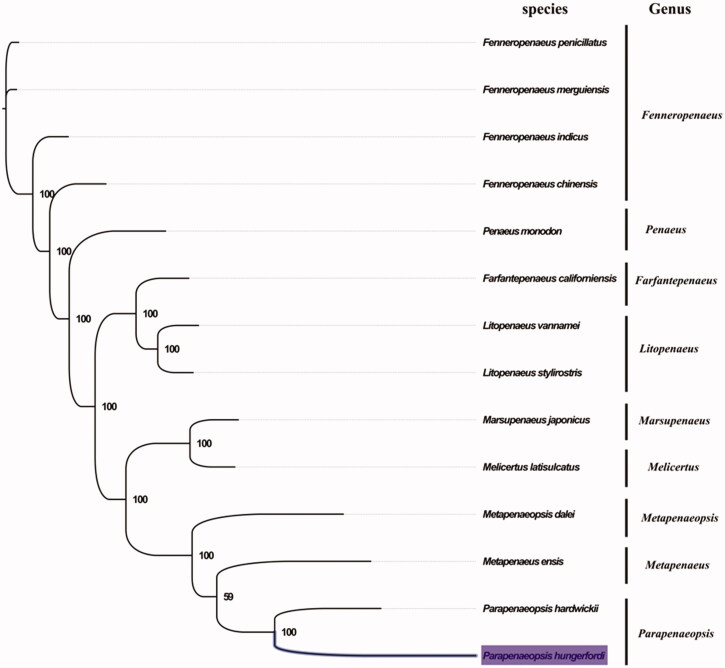
Phylogenetic tree of 14 species in family Penaeidae. The complete mitogenomes is downloaded from GenBank and the phylogenic tree is constructed by maximum-likelihood method with 100 bootstrap replicates. The bootstrap values were labeled at each branch nodes. The gene's accession number for tree construction is listed as follows: *Fenneropenaeus penicillatus* (NC_026885), *Fenneropenaeus merguiensis* (NC_026884), *Fenneropenaeus indicus* (NC_031366), *Fenneropenaeus chinensis* (NC_009679), *Penaeus monodon* (NC_002184), *Farfantepenaeus californiensis* (NC_012738), *Litopenaeus vannamei* (NC_009626), *Litopenaeus stylirostris* (NC_012060), *Marsupenaeus japonicus* (NC_007010), *Melicertus latisulcatus* (MG821353), *Metapenaeopsis dalei* (NC_029457), *Metapenaeus ensis* (NC_026834), and *Parapenaeopsis hardwickii* (NC_030277).
